# Complicated urachal cyst in two pediatric patients: a case report

**DOI:** 10.1186/s12887-023-03962-x

**Published:** 2023-03-31

**Authors:** Karol Kamel, Hadeer Nasr, Sherifa Tawfik, Ahmed Azzam, Mohamed Elsaid, Mohamed Qinawy, Ahmed Kamal, Heba Taher

**Affiliations:** 1 Department of Pediatric Surgery, Cairo University, Cairo, 11441 Egypt; 2 Faculty of Medicine, Misr University for Science and Technology, 6th of October, Giza Egypt

**Keywords:** Urachal cyst, Acute abdominal pain, Urachal anomalies

## Abstract

**Background:**

A urachal cyst has a rare incidence that has been reported as 1/5,000 live birth.

**Case presentation:**

We report two patients with a complicated urachal cyst, a 5-year-old female who presented to the emergency department with severe abdominal pain and a 3-year-old female presenting with abdominal pain and constipation. Upon laparoscopic exploration both patients had complicated urachal cysts which were adherent to the urinary bladder.

**Conclusion:**

Complicated urachal cysts can present with acute abdominal pain.

## Background

A urachal cyst has a rare incidence that has been reported as 1/5,000 live births [1]. On the other hand Physical presentation of severe abdominal pain is common in pediatric patients and approximately 10–30% of those presenting to the Emergency Department (ED) will need surgical intervention [2]. Symptoms and signs of acute abdomen indicating a higher likelihood of surgical cause are bilious vomiting, bloody diarrhea, rebound tenderness, guarding, and absent bowel sounds. Though the differential for an acute abdomen is extensive, a complicated urachal cyst is rarely ever included, and even more rarely to be observed. In the case of these patients, we are reporting a presentation with severe abdominal pain persisting for multiple days followed by extensive examination and investigations, which resulted in the finding of rare complicated urachal cysts.

## Case presentation

### Case 1

A 5-year-old girl presented to the emergency department with severe persistent abdominal pain. Her physical examination revealed pain and tenderness below the umbilicus.

**Fig. 1:**
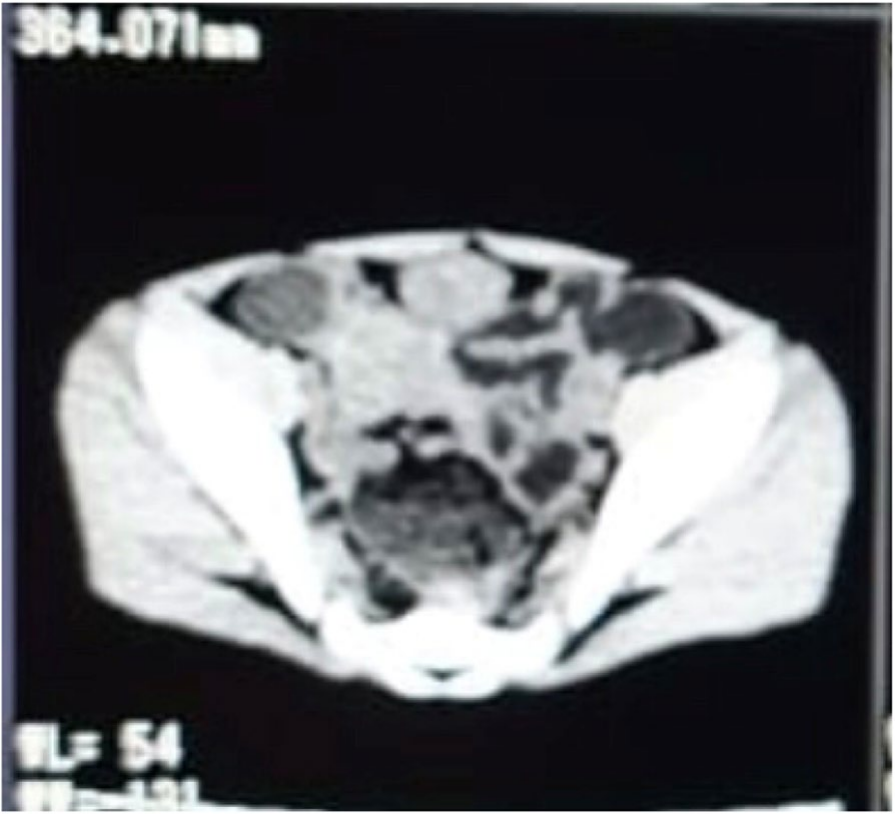
Computed tomography (CT) scan of the abdomen and pelvis showing a thick-walled cyst in the lower abdomen

Her abdominal ultrasound showed a picture of a well-defined thick-walled intraperitoneal cystic lesion in the supra-pubic region. The cyst measured 41 × 38 × 29 mm in its maximum diameters, contained dependent debris level, and was surrounded by multiple mildly thickened bowel loops suggestive of complicated duplication, mesenteric cyst, or even infected torsion of ovarian cyst. Computed tomography (CT) scan of the abdomen and pelvis with IV and oral contrast was performed and revealed a thick-walled cyst in the lower abdomen as seen in Fig. 1. Patients was put on IV fluids and first line antibiotics ciprofloxacine and metronidazole.

Laparoscopic exploration revealed a normal appendix and ovaries. Extraperitoneal swelling and inflamed omentum adherent to the small bowel and pyogenic membranes led to conversion to open exploration. No mesenteric cyst or duplication were noted.

**Fig. 2:**
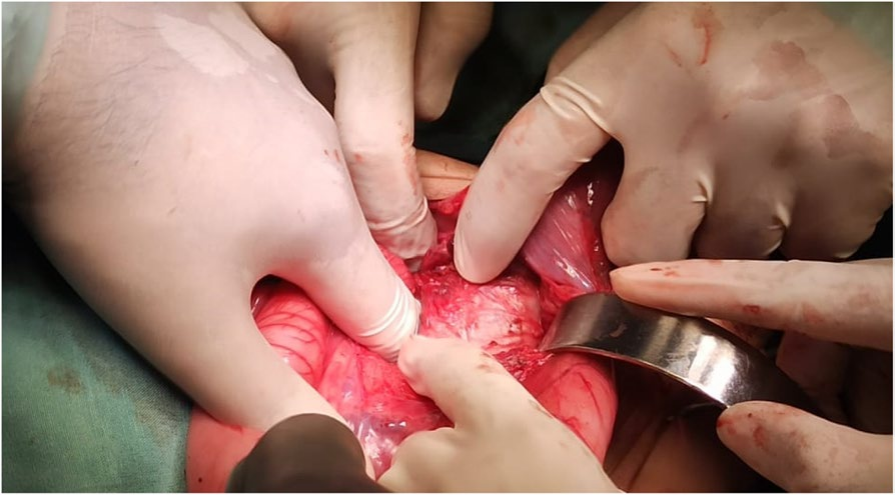
Thick-walled urachal cyst adherent to bladder fundus

Pathology was an infected thick-walled cyst filled with pus (complicated urachal cyst), adherent to the anterior abdominal wall and connected to obliterated urachus as well as being firmly adherent to bladder fundus as seen in Fig. 2.

**Fig. 3:**
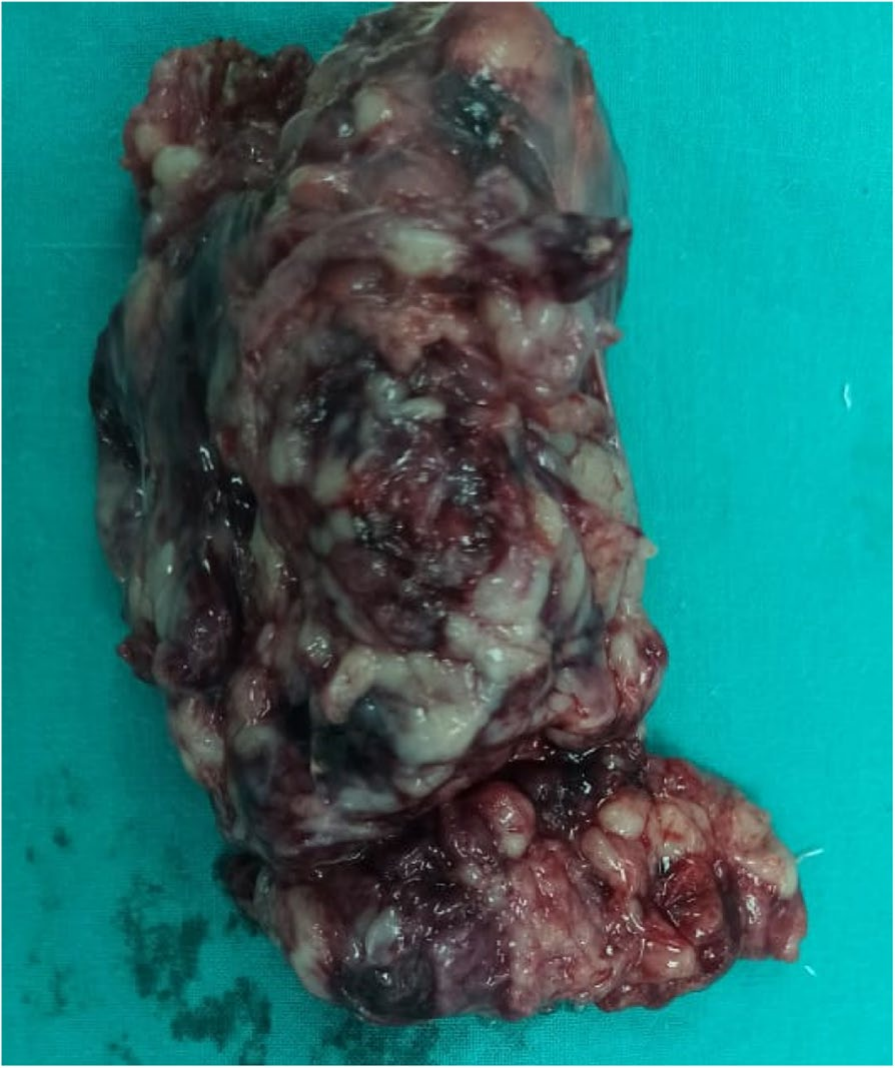
Urachal cyst excised from Case 1

We performed a complete excision of the cyst as well as the involved bladder. Afterwards, we repaired the urinary bladder using 3/0 Vicryl in two layers. Then, a 12 French urinary catheter was placed. The excised urachal cyst is shown in Fig. 3. On the fourth post operative day the urinary Catheter was removed and when the patient passed urine and discharged the following day.

### Case 2

The second patient was a 3.5-year-old pediatric female patient who presented to the emergency department with severe abdominal pain, abdominal distention, as well as constipation, and vomiting. A general examination was performed revealing a generally ill appearance with body temperature of 38℃. Her abdomen was moderately distended, lax and tender upon palpation around the umbilicus in addition to a mass that was felt infra-umbilical.

Following admission, the patient was resuscitated placing a catheter and administering intravenous bolus fluids 20 ml/ kg until urine output established 1.5 ml/kg urine output followed by administration of maintenance fluids at the same time IV antibiotics ceprofloxacin, gentamicin and metronidazol. Lab findings were relevant for a total leukocytic count TLC of 11.6. Abdominal postro-anterior view erect x-ray showed moderate dilated bowel loops with an air-fluid level with no specific findings. An abdominal pelvic ultrasound was carried out showing a cyst filled with turbid fluid related to the urinary bladder and no abnormalities of urinary tract. This was followed by a CT scan of the abdomen and pelvis with oral and IV contrast which showed patent dilated urachus extending down to the superior aspect of the urinary bladder with inflammatory process measuring 3.8 × 2.9 × 2.7 cm as well as ileo-ileal intussusception.

Upon exploration, there was no evidence of intussuception as stated in CT and an infected urachal cyst was found between the umbilicus and the urinary bladder, but it was not connected to the bladder. Evacuation and excision of the cyst were done and it was separated from the bladder. Urinary bladder mucosa was intact Repair of sero-musculosa of the urinary was performed in two layers of 3/0 Vicryl with insertion of a 12 French urinary catheter. The patients were discharged with a scheduled follow-up in the outpatient clinic. The patient was admitted for 6 days post-operatively till clinical improvement.

The specimens from both patients were sent to pathology and shown no evidence of malignancy and confirmed inflammatory nature of the specimens.

## Discussion and conclusion

There are five subtypes of congenital urachal anomalies: patent urachus, umbilical-urachal sinus, vesicourachal diverticulum, urachal cyst, and alternating sinus [3]. A urachal cyst is defined as a sac-like pocket of tissue that develops within the urachus, a canal from the bladder to the umbilicus of a developing fetus. Urachal remnants result from incomplete regression of intra-embryonic connection of the allantois to the cloaca [4]. On about day 16, the allantois appears as a finger-like projection with the ventral cloaca at one end and the umbilicus at the other. The ventral portion of the cloaca eventually develops into the bladder following cloacal division by the urogenital septum. When exactly the urachus closes is uncertain but it is thought that once the bladder descends into the pelvis during the fourth or fifth month of gestation, the urachus narrows and eventually obliterates to a thin fibrous cord in postnatal life to become the median umbilical ligament [5].

Previous literature showed that when a cyst develops on this remnant, it seldom causes any symptoms unless it is accompanied by a secondary infection. The majority of patent urachal findings are incidental when patients present with other problems as they are frequently asymptomatic. Urachal anomalies were reported to be most common in infants between the ages of 1 day to 2 years of age and the incidence rate in males was found to be twice that of females [6]. And when occurring in males posterior urethral valves or causes of bladder outlet obstruction should be investigated [4] and the ideal investigations is micturating cystourethrogram. Moreover, other urinary tract anomalies should be investigated such as vesicoureteral reflux [4], in our patients according to ultrasound sound reports and follow up there were no associated urinary tract anomalies.

The presentation of these anomalies varies drastically in the clinical setting. Urachal cysts may present as an abdominal mass and develop into an abscess if infection occurs. Symptoms then may range from generalized abdominal pain, fever, to periumbilical pain, redness, or drainage [7]. Rather, the presentation of a urachal cyst is nonspecific and presents similar to appendicitis, bowel obstruction, as in our second case, celiac disease, or inflammatory bowel disease (IBD). A thorough history, physical examination, and proper imaging are needed to diagnose urachal anomalies [8]. And resuscitative measures in infected complicated cases. An underlying condition that lowers immunity should be investigated such as diabetes mellitus. In our two patient they didn’t have an underlying debilitating conditions [9].

In order to diagnose the urachal cysts in our patients, abdominal ultrasound and CT scan were needed. Once a thick-walled cyst was noted on CT, a laparoscopic exploration was performed, which is noted in the literature to be the preferred method of exploration as it is a minimally invasive, safe, and effective method, with a better cosmetic outcome and ideal for pelvic exploration [10–14]. However, due to complications of both cases, there became a need for open exploration and surgical removal of the cyst. With advances in artificial intelligence in the surgical field, particularly in the area of radionics, better preoperative diagnosis can be achieved allowing for improved operative planning and excision of the anomaly [13, 15–18].

These cases show the presence of urachal anomalies, which could be patent, cyst, or sinus, and is often not associated with signs or symptoms needing intervention. In the case of complications, such as infection of the urachal cyst, surgical intervention is indicated. Previous studies found that only those presenting with complicated urachal cysts, larger-sized cysts, or cysts with no signs of regression should undergo surgical excision through laparoscopic means [12–14]. However, this case report highlights that in the presence of urachal anomalies, general surgeons should anticipate adherence to the bladder and be familiar with handling this structure anatomy. Excision of the wall might be needed as in the first patient or partial resections as in the second patient, and insertion of urinary catheters until the bladder wall heals. It should also be noted that nutritional assessment plays an important role in postoperative outcomes and is a crucial component of pediatric surgical patient management [19, 20].

## Data Availability

Data and materials are available upon request from the corresponding author.

## Abbreviations

CT: Computed tomography
TLC: Total leucocytic count
